# Utility of Ganglion Cell Complex Analysis in Early Diagnosis and Monitoring of Glaucoma using a Different Spectral Domain Optical Coherence Tomography

**DOI:** 10.5005/jp-journals-10008-1171

**Published:** 2015-01-15

**Authors:** Purvi Raj Bhagat, Kalyani Vivek Deshpande, Bhagyashree Natu

**Affiliations:** Associate Professor, Department of Glaucoma, M and J Western Regional Institute of Ophthalmology, Ahmedabad, Gujarat, India; Senior Resident, Department of Glaucoma, M and J Western Regional Institute of Ophthalmology, Ahmedabad, Gujarat, India; Resident, Department of Glaucoma, M and J Western Regional Institute of Ophthalmology, Ahmedabad, Gujarat, India

**Keywords:** GCC, OCT, RNFL, Pre-perimetric glaucoma, Peri-metric glaucoma.

## Abstract

**Purpose:** To determine the importance of ganglion cell complex (GCC) analysis as a parameter for early diagnosis of glaucoma and for following glaucoma progression and to compare glaucoma progression with conventional visual field analysis using a different type of spectral-domain optical coherence tomography (SD-OCT).

**Materials and methods:** Two hundred eyes including 68 normal eyes, 70 eyes with pre-perimetric glaucoma and 62 eyes with perimetric glaucoma were analyzed in this prospective study undertaken during Jan 2013 to Dec 2013 in a tertiary ophthalmology institute. Automated visual field examination was done to group the subjects in above three categories. The thicknesses of the GCC and retinal nerve fiber layer (pRNFL) were measured using Topcon model 2000 version 7.1 SD-OCT images and compared. The statistical analysis was carried out by z-test.

**Results:** The average GCC was thickest in the normal group and the thickness decreased as the severity of glaucoma increased. The mean macular GCC at the start and end of the study in pre-perimetric (94.86 ± 8.31, 90.74 ± 8.46) and perimetric (82.48 ± 13.21, 79.80 ± 12.88) eyes was lower than those in normals (102.70 ± 7.19, 101.82 ± 7.42).

**Conclusion:** Majority of the studies done on GCC analysis have used the Cirrus OCT (Zeiss). Our study has used the Topcon model 2000 version 7.1 to show that irrespective of the machine used, GCC analysis definitely plays an important role. To detect pre-perimetric glaucoma and may show progression earlier than pRNFL in pre-perimetric glaucoma.

**How to cite this article:** Bhagat PR, Deshpande KV, Natu B. Utility of Ganglion Cell Complex Analysis in Early Diagnosis and Monitoring of Glaucoma using a Different Spectral Domain Optical Coherence Tomography. J Curr Glaucoma Pract 2014;8(3):101-106.

## INTRODUCTION

Glaucoma is an irreversible and progressive optic neuropathy resulting in a characteristic visual field (VF) loss.^1^ It is the leading cause of irreversible blindness and second leading cause of blindness worldwide.^2^ There are approximately 11.2 million people aged 40 years and older with glaucoma in India and primary open angle glaucoma is estimated to affect 6.48 million persons.^3^ Although standard white on white perimetry has been considered the gold standard in documenting damage and monitoring progression, 50% of the retinal nerve fiber layer may be lost before a defect is apparent on the visual field.^4^ When correlating retinal ganglion cell atrophy with automated perimetry in glaucoma patients, a 20% loss of cells, especially large ganglion cells in the central 30° of the retina, correlated with a 5-dB sensitivity loss, while a 40% loss corresponded with a 10-dB decrease. Ten percent or fewer axons may remain by the stage of severe field loss.^5^ Considering the chronic, progressive, irreversible nature of glaucomatous damage and its global burden, it becomes important to detect glaucoma in its pre-perimetric stage and monitor disease progression.

Optical coherence tomography (OCT) is a noninvasive, objective, reproducible versatile tool based on the principle of low coherence interferometry.^6^ Recent reports have shown that spectral domain optical coherence tomography (SD-OCT) can measure peripapillary retinal nerve fiber layer (RNFL) thickness and can be useful in detecting glaucoma in the pre-perimetric stage.^7-9^ The advent of SD-OCT has renewed interest in the potential uses of macular imaging in glaucoma due to its ability to segment and measure individual retinal layers better. Retinal ganglion cells encompass three layers in the retina: the inner-plexiform layer (IPL) made up of the ganglion cell dendrites, the ganglion cell layer (GCL) made up of the ganglion cell bodies and the RNFL made up of the ganglion cell axons. All three layers, collectively known as the ganglion cell complex (GCC), become thinner as the ganglion cells die from glaucoma, making it an ideal site for imaging and detecting glaucoma progression early.^10-12^ The reduction in macular thickness has been attributed to loss of retinal ganglion cells and retinal nerve fibers.^13-15^ Ishikawa et al^16^ had indicated that the thickness of the macular nerve fiber layer and the inner retinal complex could be used to discriminate normal eyes from glaucomatous eyes. Tan et al^17^ showed that inner retinal layer thinning at the macula demonstrated by custom designed automatic segmentation software was apparent before VF changes. The macular region contains over 50% of all retinal ganglion cells and is an ideal region to detect early cell loss and changes overtime because of the high density of cells.

**Fig. 1:**
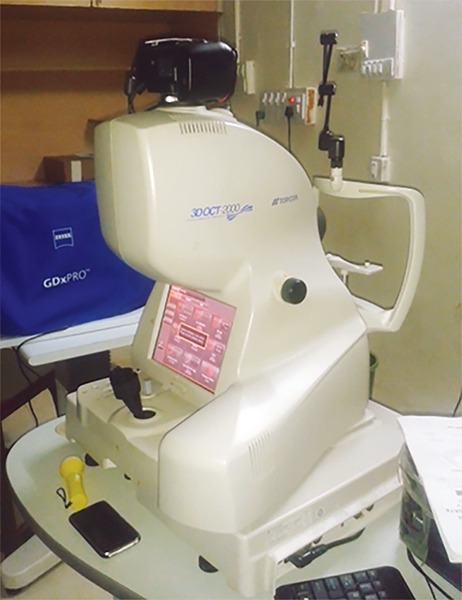
Topcon model 2000 version 7.1 (actual model installed)

This study was carried out to assess the current utility of macular SD-OCT by Topcon model 2000 version 7.1 (Fig. 1) in primary open angle glaucoma (POAG) using GCC analysis in diagnosing preperimetric glaucoma, following glaucoma progression and to compare SD-OCT parameters and VF in subjects of Western Indian population.

**Fig. 2:**
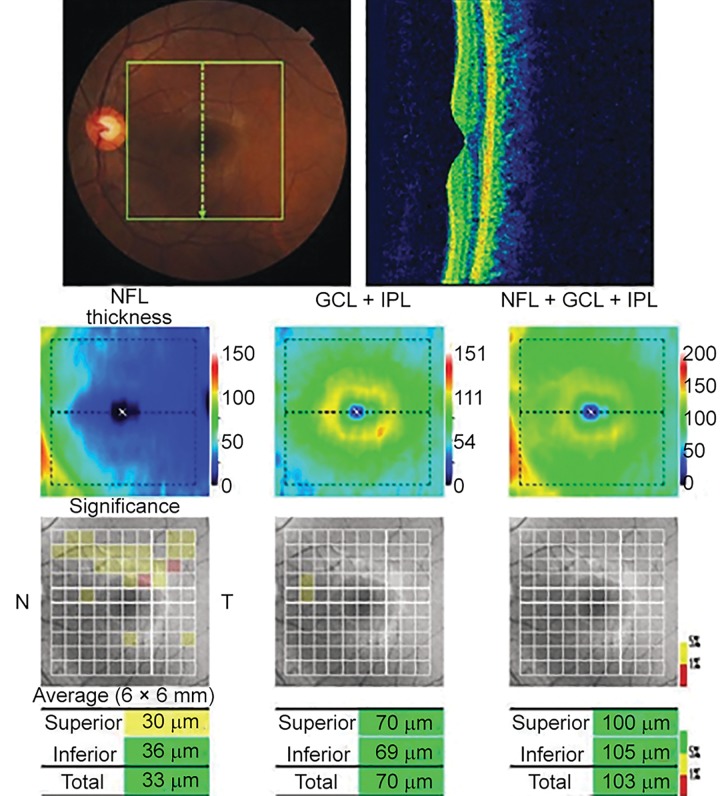
Macular scan using OCT (actual printout from Topcon model 2000 version 7.1)

## MATERIALS AND METHODS

This prospective study was conducted at a tertiary ophthalmology institute in Western India which included normal subjects and patients with POAG (perimetric and pre-perimetric). The study was undertaken during the period of Jan 2013 to Dec 2013. We studied 200 eyes including 68 normal eyes, 70 eyes with preperimetric glaucoma and 62 eyes with perimetric glaucoma. Patients with clear corneas, insignificant lenticular changes and no posterior segment pathology except disk changes suggestive of glaucoma were included. If both eyes of an individual met all criteria, a single randomly selected eye was examined. Primary open angle glaucoma patients who underwent any type of glaucomatous surgical intervention, angle closure glaucoma patients, congenital/juvenile glaucoma patients, mixed mechanism glaucoma patients, all patients with secondary glaucomas (traumatic, inflammatory, etc.) or other diseases that affect VF (e.g. pituitary tumors, demyelinating disorders). All subjects underwent detailed history taking, anterior segment examination with slit lamp, intraocular pressure measurement with Perkins’ hand held applanation tonometer, gonioscopy using Goldman two mirror goniolens and grading as per Shaffer’s classification, fundus examination using direct and indirect ophthalmoscope and slit lamp biomicroscopy by +78D lens and visual field examination with Octopus 900 static perimetry using the standard white on white tendency oriented perimetry strategy.

## GCC and RNFL Scanning Procedures

**Fig. 3:**
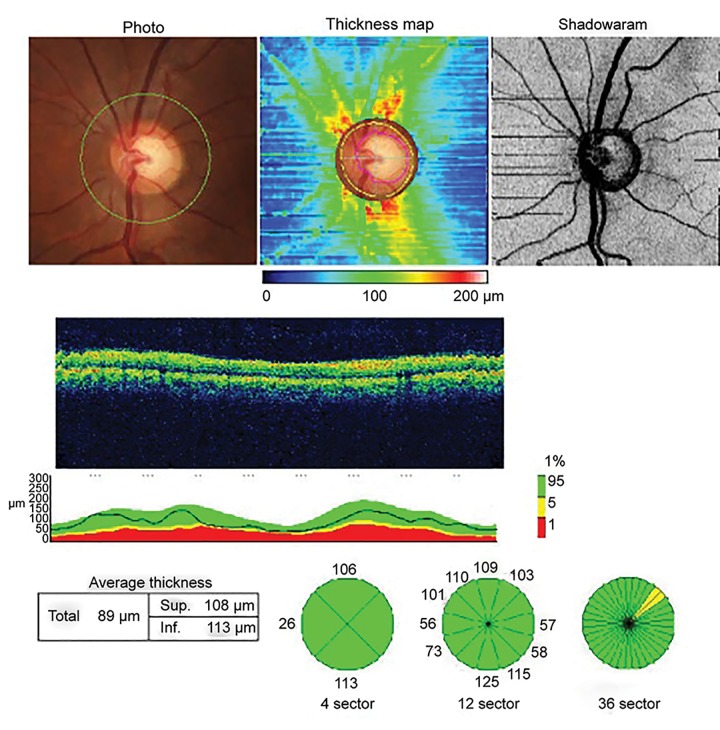
Optic nerve head scan using OCT (actual printout from Topcon model 2000 version 7.1)

Optical coherence tomography macular scan and optic nerve head (ONH) scan were performed using the Topcon SD-OCT model 2000 version 7.11 in fine analysis mode (Figs 2 and 3).

With the SD-OCT, we devised three-dimensional scans of the macular region called the GCC scan that samples the macula over a 7 × 7 mm^2^ vertical scan area in 0.6 seconds. We chose to limit the scan time to 0.6 second to reduce the problems of eye movement and corneal drying associated with long scan time. The scan pattern consists of 128 × 512 pixels taken in 7 × 7 mm^2^ area with an interval of 0.05 mm.

In ONH scan protocol, the reference plane height of disk topography is 120 microns. The pRNFL thickness was calculated as the distance between the anterior and posterior RNFL borders in a 3.45 mm radius ring centered on the optic disk. The average pRNFL thickness corresponding to the superior and inferior hemifields was measured. Well-trained operator obtained good quality images after pupillary dilatation. The criteria for determining scan quality were as follows:

1. Image quality more than 70 (according to the manufacturer).
2. A clear fundus image with clear foveal pit, ONH and scan circle before and during image acquisition.
3. Even and dense color saturation in all retinal layers, with red color visible in retinal pigment epithelium and a continuous scan pattern without missing areas.^18^

**Table 1:** **Table 1:** Characteristics of study subjects

		*N (n = 68)*		*PG (n = 70)*		*G (n = 62)*		p*		*p^#^*	
Age (years)		55.45 ± 15.42		59.21 ± 12.31		60.26 ± 11.79		0.36		0.33	
Sex (F/M)		30/38		36/34		32/30		0.33		0.34	
IOP (mm Hg)		13.28 ± 3.26		14.59 ± 3.98		13.06 ± 2.45		0.31		0.27	
SE (D)		–1.78 ± 2.98		–2.20 ± 1.59		–2.37 ± 3.06		0.07		0.11	

Data are expressed in the form of means ± SD; *Difference between normal and pre-perimetric glaucoma; ^#^Difference between normal and perimetric glaucoma; N: Normal; PG: Pre-perimetric glaucoma; G: Perimetric glaucoma; F/M: Female/male; IOP: Intraocular pressure; SE: Spherical error

**Table 2:** **Table 2:** Visual field indices over 1 year

		*Normal eyes (n = 68)*						*Pre-perimetric eyes (n = 70)*						*Perimetric eyes (n = 62)*					
*Globil* *indices*		*0 month* *(MD ± SD)*		*12 months* *(MD ± SD)*		p*		*0 month* *(MD ± SD)*		*12 months* *(MD ± SD)*		*p^#^*		*0 month* *(MD ± SD)*		*12 months* *(MD ± SD)*		p**	
MS		24.47 ± 3.25		25.31 ± 2.45		0.28		24.91 ± 2.76		25.31 ± 2.38		0.41		17.73 ± 16.86		14.59 ± 6.20		0.33	
MD		0.47 ± 1.15		0.63 ± 1.06		0.23		0.88 ± 1.86		0.63 ± 1.96		0.96		9.44 ± 5.37		9.61 ± 5.20		0.94	
sLV		1.56 ± 1.20		1.40 ± 0.43		0.21		1.86 ± 1.63		1.73 ± 1.64		0.74		6.70 ± 2.10		6.77 ± 2.03		0.89	

*VF change in normal eyes over 1 year; ^#^VF change in pre-perimetric eyes over 1 year; **VF change in perimetric eyes over 1 year

**Table 3:** **Table 3:** Thickness of pRNFL by SD-OCT

		*N (n = 68)*						*PG (n = 70)*						*P (n = 62)*					
		*0 month* *(MD ± SD)*		*12 months* *(MD ± SD)*		p*		*0 month* *(MD ± SD)*		*12 months* *(MD ± SD)*		*p^#^*		*0 month* *(MD ± SD)*		*12 months* *(MD ± SD)*		p**	
Superior pRNFL		120.44 ± 12.77		118.58 ± 12.37		0.54		113.34 ± 12.13		111.54 ± 13.27		0.91		86.58 ± 23.97		80.90 ± 26		0.35	
Inferior pRNFL		124.14 ± 10.58		121.58 ± 11.32		0.33		114.37 ± 18.85		111.94 ± 18.31		0.89		89.03 ± 27.04		84.67 ± 26		0.88	
Total pRNFL		103.44 ± 13.23		100.11 ± 18.23		0.39		94.22 ± 16.18		91.82 ± 15.55		0.52		75 ± 16.70		69.12 ± 21.95		0.23	

*pRNFL change in normal eyes over 1 year; ^#^pRNFL change in pre-perimetric eyes over 1 year; **pRNFL change in perimetric eyer over 1 year

**Table 4:** **Table 4:** Thickness of macular GCC by SD-OCT

		*N (n = 68)*						*PG (n = 70)*						*P (n = 62)*					
		*0 month* *(MD ± SD)*		*12 months* *(MD ± SD)*		p*		*0 month* *(MD ± SD)*		*12 months* *(MD ± SD)*		*p^#^*		*0 month* *(MD ± SD)*		*12 months* *(MD ± SD)*		p**	
Superior GCC		101.47 ± 11.46		101.17 ± 8.58		0.28		92.97 ± 10.20		91.20 ± 8.42		0.42		82.78 ± 12.97		79.93 ± 12.91		0.35	
Inferior GCC		103.91 ± 6.33		102.91 ± 6.31		0.23		95.85 ± 8.43		91.71 ± 8.74		0.04		83.48 ± 13.97		81.12 ± 13.45		0.84	
Total GCC		102.70 ± 7.19		101.82 ± 7.42		0.47		94.86 ± 8.31		90.74 ± 8.46		0.03		82.48 ± 13.21		79.80 ± 12.88		0.80	

*GCC change in normal eyes over 1 year; ^#^GCC change in pre-perimetric eyes over 1 year; **GCC change in perimetric eyes over 1 year

## STATISTICAL ANALYSIS

Data were analyzed for progression using z-test for statistical significance. Data were presented as the mean ± SD. For all analyses, p-values of <0.05 were considered statistically significant. The thicknesses of the GCC and the pRNFL parameters of normal eyes were compared with those of preperimetric and perimetric glaucomatous eyes by z-test.

## RESULTS

The difference in characteristics among the 4 groups was not significant (Table 1).

The mean visual field indices of subjects in all three groups did not show significant difference at the start and end of study (Table 2).

The superior, inferior and total pRNFL was normal at the start and end of 6 months and did not show statistically significant change (Table 3).

The average GCC was thickest in the normal group and the thickness decreased as the severity of glaucoma increased. The mean macular GCC at the start and end of the study in preperimetric (94.86 ± 8.31, 90.74 ± 8.46) and perimetric (82.48 ± 13.21, 79.80 ± 12.88) eyes was lower than those in normals (102.70 ± 7.19, 101.82 ± 7.42) (Table 4).

## DISCUSSION

Optical coherence tomography changes have been documented in POAG patients and suspects. Until recently, macular thickness parameters have not been commonly used in glaucoma due to results of earlier studies with time-domain OCT (TD-OCT) that revealed macular imaging to be inferior to pRNFL in the diagnosis of glaucoma.^19^

In a study conducted by Inuzuka H, Kawase K et al,^20^ they observed a significant correlation between the macular GCC of the inner or outer sector of the parafovea and in the change of the visual field in each hemifield defect or the apparently normal hemifield. The decrease of the GCC corresponding to the apparently normal hemifield correlated with the progression of the severity of the glaucomatous defects, using the Anderson classification in 67 eyes of 67 patients with open-angle glaucoma showing superior or inferior hemifield defect as measured by Humphrey field analyzer programs.

In our study, GCC thickness at the macula in a 7 × 7 mm^2^ (superior, inferior and total) in normal, pre-perimetric and perimetric eyes has been compared to visual field global indices, whereas the above mentioned study used GCC at three predetermined points and compared it with the visual field at the same points applying Anderson criteria to detect progression in established cases of perimetric glaucoma. Our study suggests potential role of GCC analysis in detecting pre-perimetric glaucoma.

In a study by Takagi ST, Kita Y et al,^21^ it was found that the thickness of the macular GCC in the normal hemifield of the glaucomatous eyes was significantly less than in normal eyes. In contrast, the total thickness of the macular retinas between the glaucomatous and normal eyes showed no significant difference. In addition, the thicknesses of the macular GCC and pRNFL in the normal hemisphere of the glaucomatous eyes significantly correlated with the total deviation in the visual field parameters of the corresponding area.

Our study similar to this study validates the role of GCC analysis in addition to pRNFL but in preperimetric glaucoma. It also concludes that GCC analysis may show progression earlier than pRNFL in preperimetric glaucoma.

In a study by Na JH, Kook MS et al,^22^ they found that perimetrically normal hemifields of glaucomatous eyes had significantly lower macular GCC and pRNFL thicknesses than did the corresponding retinal regions of healthy eyes. SD-OCT may be a useful ancillary diagnostic tool for evaluation of early macular and circumpapillary structural changes in glaucomatous eyes with localized VF defects.

In our study also, the mean macular GCC at the start and end of the study in preperimetric (94.86 ± 8.31, 90.74 ± 8.46) and perimetric (82.48 ± 13.21, 79.80 ± 12.88) eyes was lower than those in normals (102.70 ± 7.19, 101.82 ± 7.42). Our study compared OCT parameters with the global indices of visual field and not with the affected hemi-retinae.

In a study by Moreno PA, Konno B et al,^23^ they found that average mean deviation was -2.5 ± 1.6 dB for the glaucomatous eyes. The area under curve (AUC) for average, superior and inferior macular inner retinal thicknesses was not significantly different (p ≥ 0.18). The AUCs for average, superior and inferior pRNFL thicknesses were also similar (p ≥ 0.15). Average macular inner retinal thicknesses had a significantly larger AUC compared to average pRNFL thickness analysis (0.815 *vs* 0.735; p = 0.03).

Our study similar to this study considers GCC scan to be superior to pRNFL in detecting early glaucoma.

In a study by Rao HL, Zangwill LM et al,^24^ RTVue RNFL and inner retinal macular thickness measurements had good ability to distinguish eyes with glaucomatous visual field loss and performed significantly better than ONH parameters.

This conclusion is similar to our study which proved macular screening by GCC analysis to be better than ONH measurements. This study compares macular RNFL with GCL/IPL parameters (both macular parameters) whereas our study compared GCC analysis with pRNFL measurements.

In a study by Mwanza JC, Durbin MK et al,^25^ they found that the ability of macular GCIPL parameters to discriminate normal eyes and eyes with early glaucoma is high and comparable to that of the best pRNFL and ONH parameters.

Our study found GCC superior to pRNFL in determining eyes with early glaucoma.

Single baseline measurements of GCC layer in glaucomatous and nonglaucomatous eyes also show significant difference (p < 0.001 for superior, inferior and total GCC values in normal eyes compared to glaucomatous eyes). The total GCC values in glauco-matous eyes compared to normal eyes showed very significant statistical difference.

Most of the studies on macular GCC have been carried out on Cirrus SD-OCT. However our study differs in this respect as Topcon SD-OCT assesses the GCC in a different way but gives similar results. One of the limitations of our study is its small sample size and the fact that our study was carried out over small period of time. However the results being significant, definitely yield important information. Also we have taken single OCT measurement at one time point, though earlier studies support the fact that the reliability and reproducibility of single OCT measurements is good. Yet it needs to be determined if increasing the number of OCT measurements can yield better results.^26,27^

To conclude, the assessment of GCC parameters plays an important role in diagnosis and monitoring of glaucoma and all models of SD-OCT can be equivalently used to producing similar results.
